# Metabolic Flux and Growth Profiling of *Megasphaera cerevisiae* for Medium‐Chain Fatty Acid Synthesis

**DOI:** 10.1111/1758-2229.70390

**Published:** 2026-07-24

**Authors:** Wael Sabra, Sonia Villotti, Joachim Fensterle, An‐Ping Zeng, Ahmed M. Haddad

**Affiliations:** ^1^ Faculty of Life Sciences Rhine‐Waal University of Applied Sciences Kleve Germany; ^2^ Center of Synthetic Biology and Integrated Bioengineering, School of Engineering Westlake University Hangzhou China; ^3^ Environmental Biotechnology Department, Genetic Engineering and Biotechnology Research Institute City of Scientific Research and Technological Applications (SRTA‐City) Alexandria Egypt

## Abstract

*Megasphaera cerevisiae* is a well‐known beer spoilage organism, capable of producing undesirable flavours and turbidity. Although the biosynthesis of medium‐chain fatty acids (MCFAs) has been extensively studied in different Megasphaera species, the metabolic behaviour of *M. cerevisiae* in controlled environments remains largely unexplored. This study examines the MCFAs production from diverse substrates and reports flux analyses of core metabolism for the first time using a genome‐scale model. The results suggest that acetate stimulates butyrate production but not caproic acid production. Butyrate supplementation, either alone or in combination with acetate, promoted CA synthesis. Lactate supplementation primarily led to the formation of propionic acid and acetic acid. The metabolic network model was manually curated and validated against the different experimental data. The metabolic flux results showed that butyrate production facilitated via the reverse β‐oxidation (RBO) pathway, with a minor contribution from the fatty acid synthesis (FAS) pathway. Conversely, CA synthesis was mainly synthesised through the FAS pathway, irrespective of the substrate used. The pathway analyses results highlighted the critical role of hydrogen production in *M. cerevisiae* metabolism, particularly under conditions where lactate is utilised. Collectively, these findings offer novel insights into the metabolic versatility and pathway preferences of *M. cerevisiae* .

## Graphical abstract

Characterising the metabolism of medium‐chain fatty acids in *Megasphaera cerevisiae* , based on genome‐scale metabolic modelling and integration with experimental data obtained from controlled bioreactor cultivations. The iterative approach aligns in silico predictions with empirical observations and a refined model was obtained.

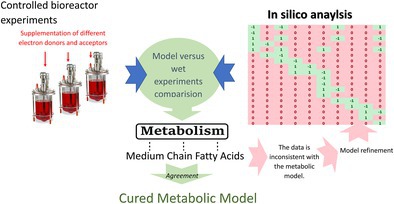

## 1 Introduction

Caproic acid (CA), also known as hexanoic acid, is a versatile organic compound with significant importance in various industries and biotechnological applications. This six‐carbon fatty acid plays a crucial role in both natural processes and synthetic applications, making it a valuable target for research and industrial production. In fact, the microbial production of CA did not become evident until the first isolation of Clostridium kluyveri, and its production and the mechanism of its synthesis were investigated from then on (Bornstein and Barker 1948). A number of approaches were evaluated for their potential to produce CA using biotechnology. One approach that shows considerable promise is the use of mixed organic waste as a feedstock in a biorefinery process (Bian et al. 2024). However, limitations such as the inherently unstable nature of the biological process, fluctuations in the structure of the microbiome, and the existence of competitive metabolic pathways have consequently diminished the economic viability of the CA process using such an approach. A more simplified co‐culture system was also used to produce CA via lactate using Acetobacterium woodii and Clostridium drakei (Herzog, Mook, Guhl, et al. 2023; Herzog, Mook, Utesch, et al. 2023). Compared to mixed and co‐culture fermentation, pure culture fermentation offers a more streamlined process, making it easier to define experimental variables and explore microbial metabolic pathways (Kenealy et al. 1995).

The synthesis of CA and MCFAs, in general, is facilitated by two primary metabolic pathways: the reverse β‐oxidation (RBO) and the fatty acid biosynthesis (FAS) pathway. Acyl carrier protein (ACP) is a key metabolite of the FAS pathway and is found in all bacterial strains (Cronan and Thomas 2009). The RBO pathway involves elongating acetate/n‐butyrate by two carbons at a time and converting them into chemicals with six or more carbons in sequence (Dahiya and Mohan 2023). This pathway requires substrates containing both electron donors and electron acceptors, such as short chain fatty acids (Liu et al. 2024). In addition to *Clostridium kluyveri* , the first strain identified as being capable of producing CA, numerous other bacterial species have recently been recognised as promising candidates for the biotechnological synthesis of CA from various substrates (Jeon et al. 2016; Kim et al. 2020, 2; Wang et al. 2018). A multitude of compounds have been used as electron acceptors with pure bacterial cultures, including acetate, propionate, *n*‐butyrate, as well as the dicarboxylates succinate and malate. Lactate has also been described as an electron donor to generate the acetyl‐CoA to provide the two carbon atoms for the acetate to *n*‐butyrate elongation via RBO (Wang et al. 2018; Lu et al. 2021; Andersen et al. 2015).

Different strains of *Megasphaera* were studied for the production of MCFAs. Among others, *M. elsdenii* , *M. hexanoica*, *M. indica, M*.sp. MH were investigated on different substrates (Weimer and Moen 2013; Kang et al. 2022; Prabhu et al. 2012; Jeon et al. 2016; Lanjekar et al. 2014). RBO pathway and chain elongation with lactic acid have been described in *Megasphaera elsdenii* (Spirito et al. 2014; Angenent et al. 2016). Moreover, *Megasphaera* sp. MH achieved a high titre of 9.7 g/L of caproic acid within 24 h (Jeon et al. 2016). *M. hexanoica* has also demonstrated efficient caproic acid production using lactate as the sole electron donor (Kang et al. 2022). *Megasphaera cerevisiae* strains are responsible for beer spoilage, producing undesirable flavours and aromas including acetoin and various acids such as butyric, acetic, isovaleric, and caproic acids (Engelmann and Weiss 1985). It has been recorded that *M. cerevisiae* contains numerous transcripts associated with lactate utilisation, including L‐lactate dehydrogenase and l‐lactate permease (Bergsveinson et al. 2017). While these enzymes are necessary for acetate formation, they may be involved in chain elongation to butyrate and CA production. To date, no systematic studies have explored the maximum production of MCFA in controlled bioreactors or characterised the key growth parameters of *M. cerevisiae* . Furthermore, the utilisation of lactate as an electron donor, analogous to its function in the closely related *M. elsdenii* , remains unreported. Despite the genome of *M. cerevisiae* being available, its use for metabolic flux or pathway analysis remains to be explored. This study aims to examine the impact of cultural conditions on the growth, substrate utilisation, and product formation processes in *M. cerevisiae* . The different metabolic pathways involved in the processing of various electron donors are also analysed and genome scale metabolic flux analysis (MFA) is conducted.

## 2 Material and Methods

### 2.1 Bacterial Strain, Culture Medium and Growth Conditions

*Megasphaera cerevisiae* DSMZ 20462 was cultivated anaerobically at 35°C without shaking. For seed culture preparation, the PYF complex medium containing the following ingredients in 1 L of distilled water was used: Tryptone, 5 g; Peptone, 5 g; Yeast extract, 10 g; Fructose, 10 g; K_2_HPO_4_, 2 g; Resazurin, 1 mg; Cysteine‐HCl. H_2_O, 0.5 g; Haemin solution, 10 mL; Vitamin K1 solution, 0.2 mL; Mineral solution, 40 mL. The haemin solution was prepared by dissolving 50 mg haemin in 1 mL 1 N NaOH; the solution was completed to 100 mL with distilled water and was stored refrigerated. The Vitamin K1 solution was prepared by dissolving 0.1 mL of vitamin K1 in 20 mL of 95% ethanol, and it was sterilised by filtration and stored refrigerated in a brown bottle. The Mineral solution contained the following ingredients in 1 L of distilled water: CaCl_2_.2H_2_O, 0.25 g; MgSO_4_.7H_2_O, 0.5 g; K_2_HPO_4_, 1 g. KH_2_PO_4_, 1 g; NaHCO_3_, 10 g; NaCl, 2 g. The pH was adjusted to 7.2 using 8 N NaOH, and the medium was boiled and cooled under nitrogen to ensure anaerobic conditions and autoclaved. The vitamin K1 and haemin solution were added after the medium had been boiled and cooled. After inoculation for 1 day at 35°C, culture stocks were preserved using glycerol 20% (v/v) at −80°C.

In addition to the complex PYF media, minimal media was utilised for the cultivation of *M. cerevisiae* in controlled bioreactors. The minimal medium contained the following ingredients in 1 L of distilled water: Yeast extract, 0.5 g; Fructose, 10 g; Cysteine‐HCl. H_2_O, 0.5 g; Vitamin solution, 1 mL; Mineral solution, 40 mL. The vitamin solution contained the following in 1 L; Biotin, 0.5 mg; Pyridoxine, 20 mg; Calcium pantothenate, 20 mg. The mineral solution contained the following ingredients in 1 L of distilled water; CaCl_2_, 0.125 g; MgSO_4_.7H_2_O, 0.125 g; K_2_HPO_4_, 1 g; KH_2_PO_4_, 1 g; NaHCO_3_, 5 g; NaCl, 2 g; (NH_4_)_2_SO_4_, 2.5 g; MnSO_4_.H_2_O, 0.05 g; FeSO_4_.7H_2_O, 0.05 g; ZnSO_4_.7H_2_O, 0.05 g; CoSO_4_.6H_2_O, 0.005 g. The medium pH was adjusted to 7.0 using 8 N KOH.

Batch cultivations with either complex or minimal media were performed in a 1.5 L well‐equipped 4‐parallel bioreactor system (DASGIP Parallel Bioreactor System, Jülich, Germany) with 1.0 L initial working volume. After sterilisation, the medium in the bioreactor was flushed with sterile oxygen‐free nitrogen gas until the room temperature was reached. Fructose and different additives (butyric, acetic, and lactic acid) were autoclaved separately and were added together with the sterile cysteine HCl solution and inoculated immediately. Cultivations were initiated by inoculating 50 mL (5%) of a 48‐h‐old seed culture. The bioreactor system was equipped with a PT‐100 temperature probe, redox and pH probes from Mettler‐Toledo. Before all fermentations, the probes were calibrated according to the manufacturer instructions. pH was maintained at 7.0 during all the cultivations by the automatic addition of 5 N NaOH. Flushing with nitrogen was stopped after inoculation, and the bacteria were grown under their own produced gases.

### 2.2 Analytical Methods

Cell concentration was measured optically, at 600 nm and correlated with cell dry weight determined directly. In the fermentation experiment conducted with minimal media, the strains formed floccules, and the optical density was measured following vertexing with glass beads for 2 min. The concentrations of sugar and organic acids in the cell free supernatant were determined by HPLC using a Phenomenex HPLC (Rezex ROA‐Organic Acid H+ (8%), column (300 × 7.8 mm)) with H_2_SO_4_ 0.005 M as a mobile phase and with 0.6 mL/min flow rate for 1 h at 70°C. The detection was assessed by refractive index and ultraviolet detectors.

### 2.3 In Silico Metabolic Model Construction and Metabolic Flux Analysis

The genome sequence of *M. cerevisiae* DSM 20462 was retrieved from the public database and loaded into the Department of Energy System Biology Knowledgebase (KBase) Narrative (Arkin et al. 2018). Annotation was performed by Rapid Annotations using Subsystems Technology (RAST). Finally, for the integration of metabolic annotations and the reconstruction, comparison, and analysis of metabolic models, the ModelSEED2 Biochemistry Database was used (Seaver et al. 2020).

**FIGURE 1:**
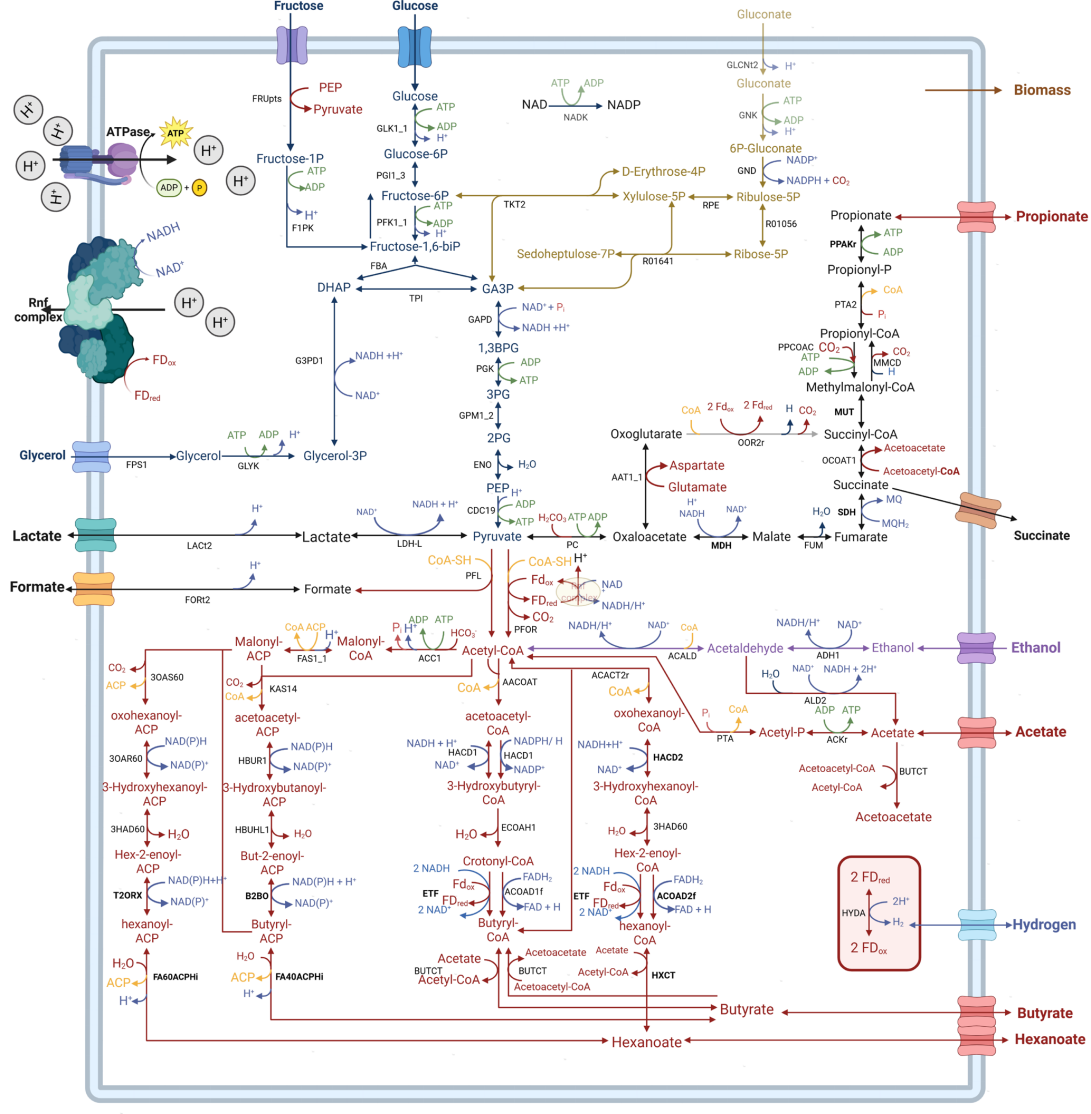
Schematic representation of the metabolic network model for fatty acid synthesis in *Megasphaera cerevisiae* (Created with BioRender.com). The core pathway comprises 109 reactions, of which 28 were exchange reactions and 11 (highlighted in bold) were incorporated through a gap‐filling process to ensure metabolic functionality.

A draft genome‐scale metabolic model of *M. cerevisiae* was first reconstructed based on glucose minimal medium, comprising 1063 reactions, 1121 metabolites, 818 genes and 94 gap filling reactions. This genome‐scale reconstruction formed the basis for the manual curation of a core metabolic network within the CNApy software environment (Thiele et al. 2022), which encapsulates the principal metabolic pathways of *M. cerevisiae* (Figure 1). According to the RAST annotation, both the FAS and RBO pathways appear to be incomplete. In the FAS pathway, for example, the final two steps are notably absent: specifically, the conversion of 2‐enoyl‐ACP to fatty acyl‐ACP (e.g., butyryl‐ACP or hexanoyl‐ACP), followed by the release of free fatty acids via thioesterase. Interestingly, a sequence in the strain studied (WP_048515320.1) shows homology to the enoyl‐ACP reductase FabI from *Escherichia coli* , which catalyses the reduction of 2‐enoyl‐ACP to fatty acyl‐ACP. However, this sequence has been annotated as FabG. Indeed, the considerable diversity of enoyl‐ACP reductases across bacterial species has previously being reported by (Massengo‐Tiassé and Cronan 2009). Additionally, an esterase/lipase family protein belonging to the GDSL type was found in *Megasphaera cerevisiae* (WP_048515163.1), and it may function as a thioesterase, as suggested by (Ji et al. 2023). Despite the apparent efficiency of RAST annotation, the prevalence of missing or inconsistent pathways in metabolic annotation has been well‐documented (Griesemer et al. 2018). The reconstruction of the model was integrated with available genomic annotations, and was supported by proteomic evidence from UniProtKB. Table S1 contains a comprehensive catalogue of the reactions included in this study. The core model encompasses all the key pathways of central carbon metabolism. This includes the uptake and utilisation of both glucose and fructose, as well as the secretion of the main products detected in laboratory experiments in mmol per g dry weight per hour (mmol/g_DW_*h). Biomass formation (growth flux) was included into the model to account for the drain of precursors and building blocks into biomass. It was assumed that the stoichiometric reaction given by Cheng et al. (Cheng et al. 2013) for the growth of *Clostridium pasteurianum* can be used.

## 3 Results

**FIGURE 2:**
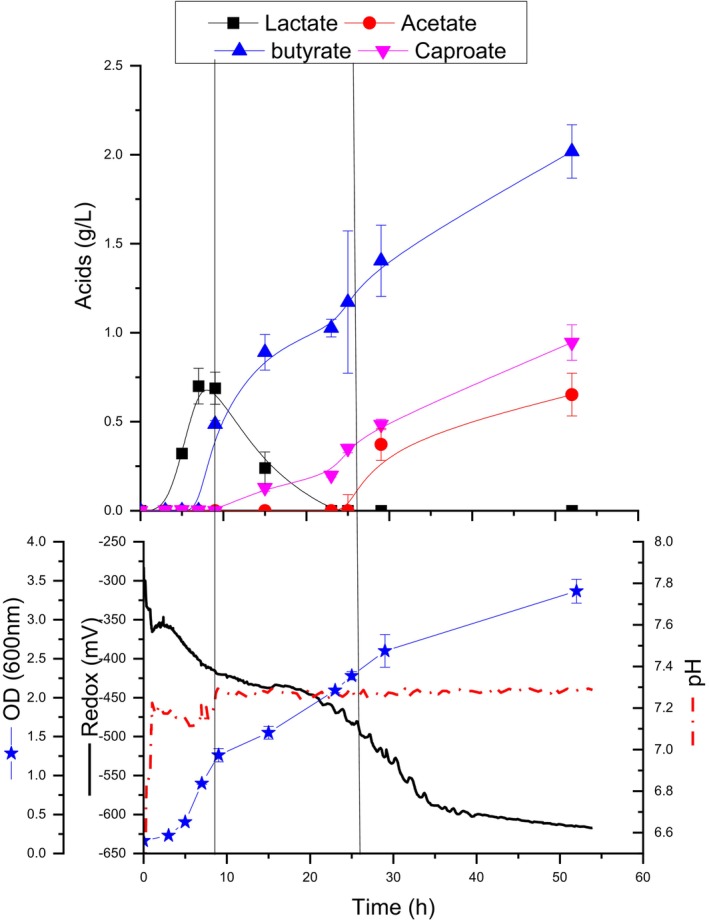
Growth kinetics and organic acid production by *M. cerevisiae* on a semi‐synthetic medium in pH‐controlled bioreactor.

**FIGURE 3:**
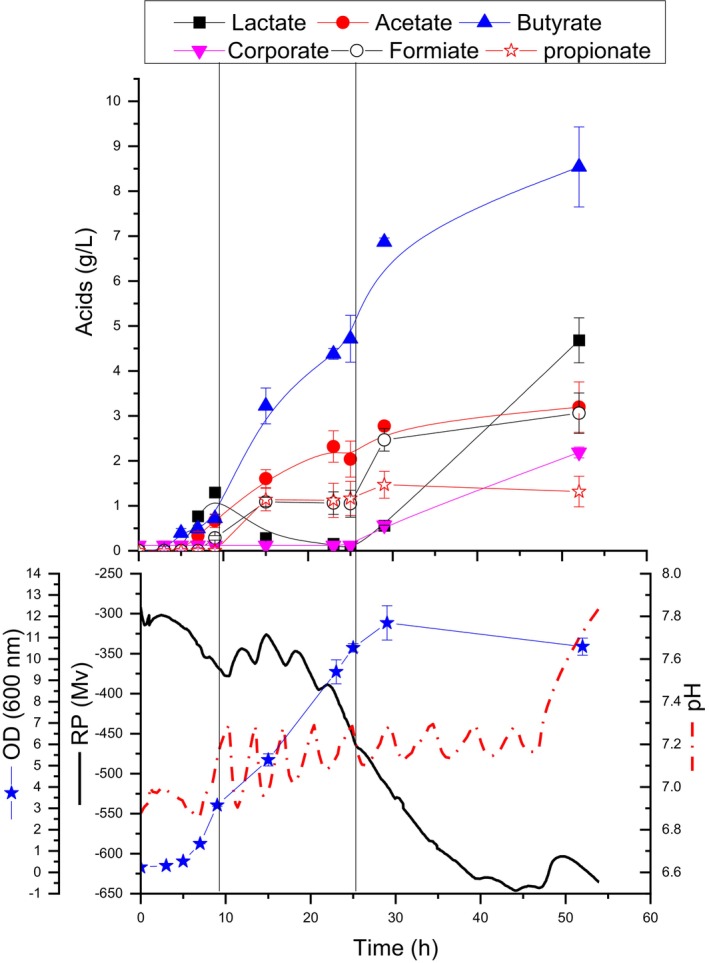
Growth and volatile fatty acids production of *M. cerevisiae* on PYF complex medium in pH‐controlled bioreactor.

In pure culture fermentation, the utilisation of diverse strains of *Megasphaera* has been documented to yield elevated concentrations of CA in media including complex nitrogen sources, such as yeast extract, tryptone, and beef extract. These complex nitrogen sources are a significant cost factor for the viability of any industrial production. A comparison was thus made of the growth and volatile fatty acid production of *M. cerevisiae* in complex and semi‐synthetic medium, with fructose as the sole carbon source (Figures 2 and 3). As expected, production of the acids increased significantly when the complex medium was used, with a total fatty acid concentration of 22.86 ± 1.2 g/L compared to 3.62 ± 0.8 g/L when the minimal medium was used. Notably, the CA content compared to the total acids produced at the end of the cultivation process was 9.5% in the complex medium and 26% in the minimal medium. Furthermore, the yield of CA from biomass was 0.83 ± 0.02 g/g and 1.12 ± 0.11 g/g in the complex and minimal media, respectively. Moreover, and regardless of the medium used, the specific growth rate of *M. cerevisiae* exhibited a clear division into three phases (Figures 2 and 3). The first phase was characterised by the highest growth rate, a sharp decrease in the redox potential and different acids production. This was followed by a second phase during which the reduction in redox potential proceeded at a slower rate. The specific growth rates observed during the second phase were at least five times lower than that recorded during the first phase. Moreover, lactate consumption is indicative of the second phase. Ultimately, a third phase with further reduction in redox potential and cessation of growth was observed. The re‐consumption of volatile fatty acids, such as lactic acid in the second phase, has been demonstrated to potentially induce chain elongation, a phenomenon that has been extensively documented in numerous bacterial strains (Andersen et al. 2015; Lu et al. 2021). Therefore, the objective of the following experiments is to ascertain the impact of incorporating diverse electron acceptors on the synthesis of CA by *M. cerevisiae* .

### 3.1 The Impact of Co‐Substrate Fermentation on the Growth and Product Profile of *Megasphaera cerevisiae*

#### 3.1.1 Metabolic Flux Analysis of *M. cerevisiae* During Growth on Acetate as an Electron Donor

**FIGURE 4:**
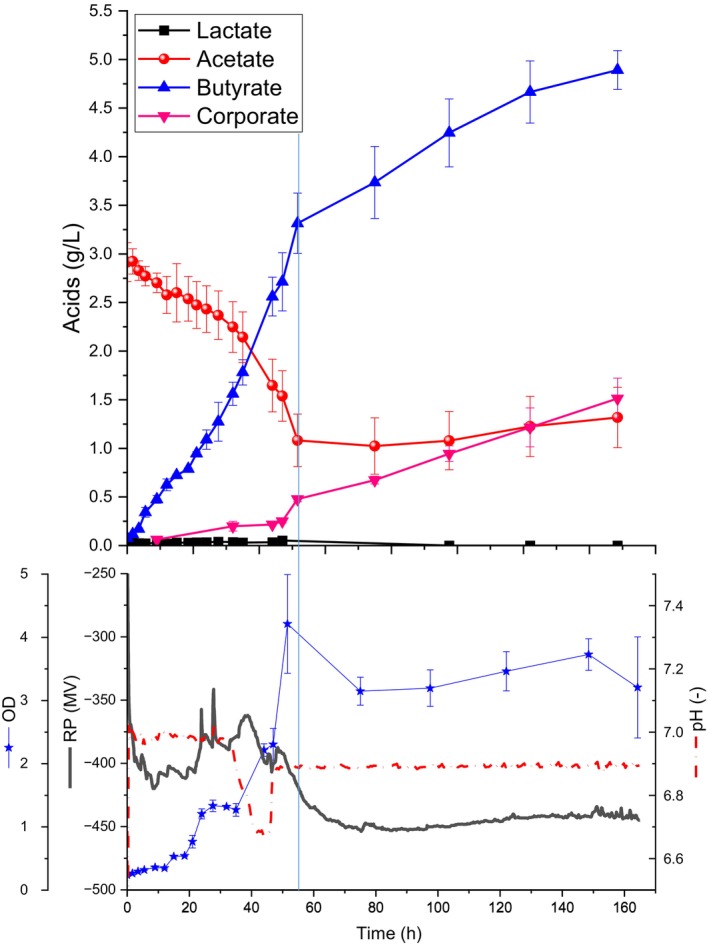
Growth and acids production of *M. cerevisiae* on semisynthetic medium supplemented with acetate in a pH‐controlled bioreactor.

**FIGURE 5:**
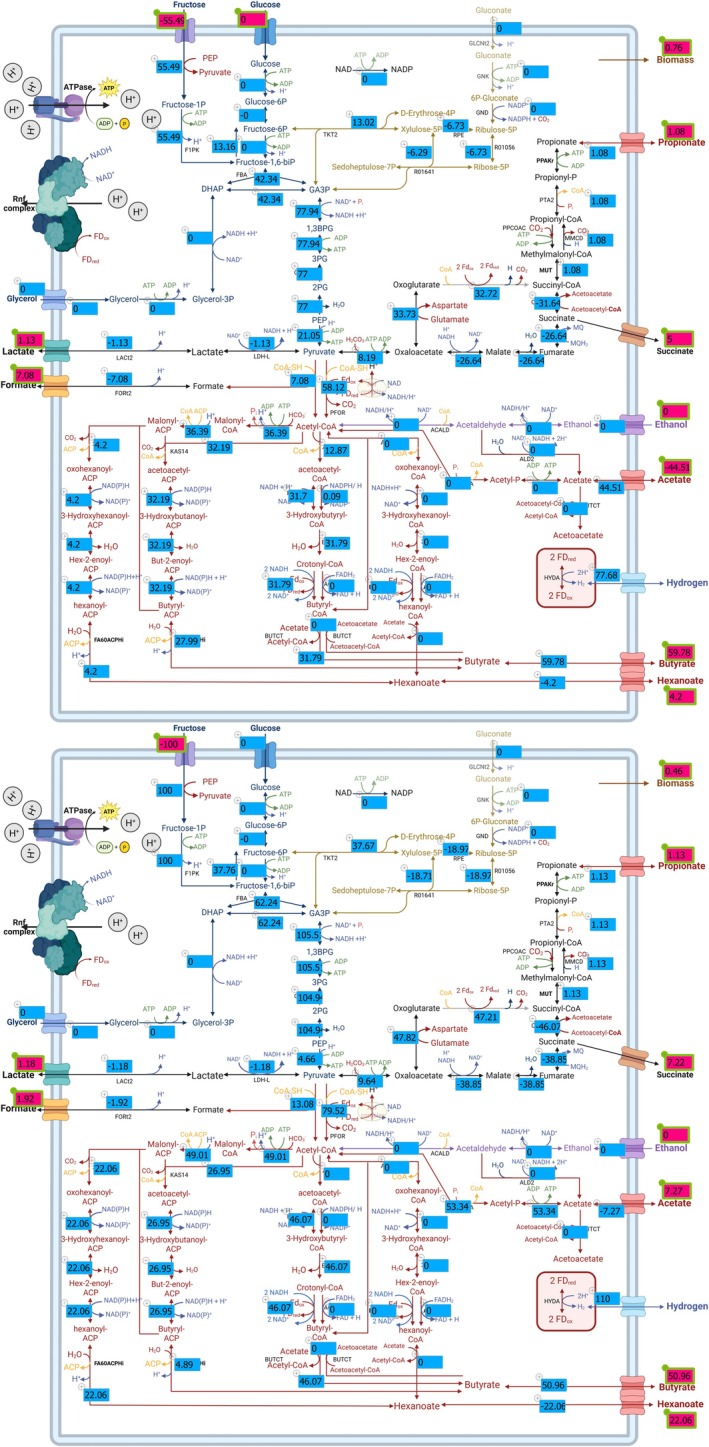
Metabolic flux analysis of *Megasphaera cerevisiae* grown on fructose and acetate. (A) In the initial phase, fructose and acetate uptake were employed as constraints for the metabolic model. (B) In the second phase, fructose uptake was utilised as a constraint for the metabolic model.

As illustrated in Figure 4, *M. cerevisiae* growth on both acetate and fructose in a semi‐synthetic medium can be divided into two distinct phases. The first phase is characterised by the consumption of acetate and fructose (0.03 and 0.12 g/L*h, respectively), accompanied by butyrate production at a rate of 0.06 g/L*h and a rapid growth rate (0.053 h^−1^). In the second phase, both growth and acetate assimilation ceased, butyrate production declined to 0.017 g/L*h, and caprate production started at a rate that was twice that observed in the first phase. It is noteworthy that lactate production was insignificant in comparison to growth on fructose as the sole carbon source (Figures 2 and 3). The experimental data from both phases depicted in Figure 4 were applied as measurements constraints to the metabolic model illustrated in Figure 1, and the metabolic model showed feasibility for both phases. The metabolic model predicted an increase in hydrogen flux from 77 to 110 mmol/g_DW_*h in the first and second growth phases, respectively. This was consistent with the measured redox potential in the thesis cultures, which decreased from −400 to −450 mV in the first and second phases, respectively (Figure 4). It is noteworthy, that in both phases, RBO and FAS pathways were found to be active, and butyrate was synthesised through both pathways, while CA was synthesised predominantly via the FAS pathway (Figure 5a).

#### 3.1.2 Triggering Caproate Production Using Butyrate as a Medium Supplement

**FIGURE 6:**
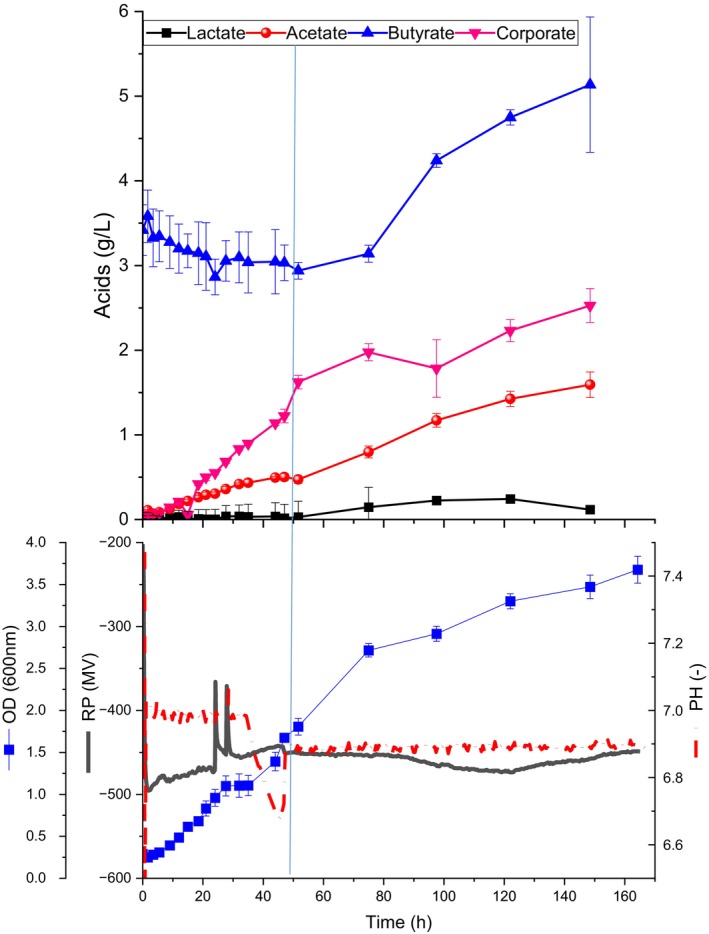
Growth and VFA production of *M. cerevisiae* on semisynthetic medium supplemented with butyrate in pH‐controlled bioreactor.

In contrast to the cultivations with acetate as a co‐substrate (Figure 4), where CA began to accumulate in the second phase when butyrate already accumulated, CA accumulation started relatively at the cultivation's beginning in bioreactors with initial butyrate addition (Figure 6). The growth phase with butyrate as the initial medium supplement is again subdivided into two phases. The initial phase exhibited a peak in CA production, with a concentration of almost 2 g per litre being reached after 75 h. This was accompanied by a slight increase in acetic acid production, while butyric acid concentration decreased slightly. In the ensuing phase, the production of CA was reduced, with an increase in butyric acid concentration being observed once more in the fermentation media. It is evident that in *M. cerevisiae* , butyric acid stimulates caproic acid production, resulting in a maximum caproate concentration of 2.5 g/L, in comparison to 1.5 g/L with acetate as a co‐substrate (Figures 4 and 6). MFA was also performed using the metabolic model shown in Figure 1, and the experimental data from both growth phases (Figure 6). The first phase was characterised by the co‐consumption of fructose and butyrate, while the second phase was characterised by the consumption of fructose only. The metabolic model predicted no change in hydrogen production between the two phases, which was shown by the negligible change in the redox potential values of the culture during the two phases (Figure 6). Moreover, the MFA results demonstrated that butyrate is synthesised exclusively by the RBO pathway, while for CA production, both the RBO and FAS pathways were found to be active in both phases, with a greater proportion of fluxes being directed towards the FAS pathway (see Figure S1). It is noteworthy that, analogous to the results observed with butyrate as a co‐substrate, the caproate production started at the initial growth phase in cultivations with both acetate and butyrate as co‐substrates (Figure S2).

#### 3.1.3 The Methylmalonyl‐CoA Pathway Directs Lactate to Propionate, Unaffected by Butyrate Supplementation for Caproate Synthesis

**FIGURE 7:**
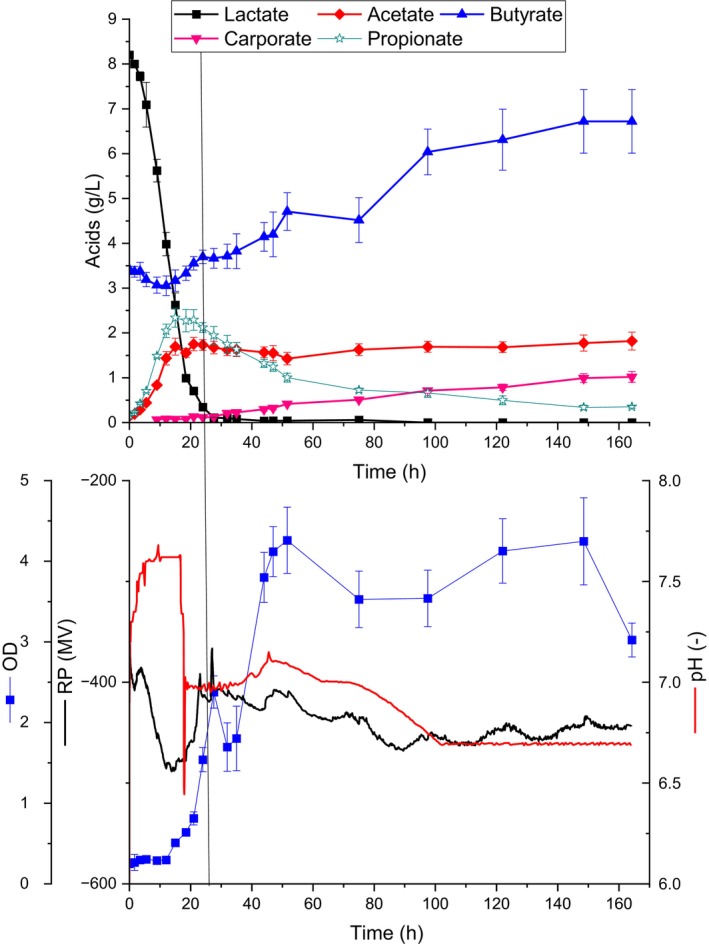
Effect of supplementing lactate and butyrate to the growth medium of *M. cerevisiae* and VFA production in controlled bioreactor.

While lactate production is associated with enhanced initial growth of *M. cerevisiae* (Figures 1 and 2), its contribution to chain elongation is unclear. To test its function as an electron donor, cultivations of *M. cerevisiae* with lactate and butyrate, a preferred electron acceptor for this species (Figure 7), were analysed. As shown in Figure 7, the addition of lactate to the medium stimulated the growth of *M. cerevisiae* , with the highest growth rate recorded on a semisynthetic medium (0.07 h^−1^). It is noteworthy that fructose, lactate, and to some extent butyrate were consumed simultaneously, with no observable diauxic growth. Despite the presence of excess fructose, growth ceased shortly after complete lactate consumption, alongside the production of propionate and acetate. Applying the measurement constraints obtained from Figure 7, on lactate and butyrate as supplements, to execute a MFA with the metabolic model shown in Figure 1 resulted in a redox imbalance and infeasibility of the model. The model was feasible only if ethanol formation occurred as an alternative electron sink, which was not consistent with the measured experimental metabolite profiles.

## 4 Discussion

Members of the *Megasphaera* species are regarded as potent beer‐spoiling anaerobic bacteria that produce a variety of short‐ and mid‐chain fatty acids (Arnold et al. 2024). Consequently, the genomes of several strains were subjected to whole genome sequencing (Bergsveinson et al. 2017; Kutumbaka et al. 2015). In this study, we examined the metabolic profile of *M. cerevisiae* DSMZ 20462 in pH‐controlled batch cultures under varied growth conditions. A genome‐scale metabolic network that describes its core metabolism was for the first time evaluated (Figure 1) under different conditions. In media containing fructose as the sole carbon source (see Figures 2 and 3), the initial fast growth phase was accompanied by lactate production, followed by a phase of lactate consumption with a low growth rate (more than five times lower). The consumption of lactate occurred despite the presence of sufficient fructose in the medium (9.2 g/L in minimal medium and 8.9 g/L in complex medium). To gain more insight about the metabolism of *M. cerevisiae* , experiments were performed using the fructose minimal media, supplemented with various carbon sources and metabolic flux analyses were performed both before and after the assimilation of supplemental electron donors.

Growth of *M. cerevisiae* in media with 3 g/L acetate resulted in a significant increase in butyric acid production. Interestingly, when acetate was being actively consumed, caproic acid levels remained negligible, suggesting that butyrate synthesis was constrained by acetate availability. Following the cessation of acetate consumption, the butyrate production rate declined from 0.06 to 0.01 g/L·h, while caproate levels began to rise gradually, reflecting a metabolic shift in carbon flux (Figure 4). While (Seedorf et al. 2008) suggested that most genes responsible for butyric acid production function in further chain elongation of MCFAs production, this hypothesis fail to explain the low rate of CA production at the first growth phase where acetate is actively consumed. In general, it does not provide a satisfactory explanation for our previous research with *Clostridium pasteurianum* , which demonstrated butyrate accumulation without detectable CA production (Schmitz et al. 2019; Utesch et al. 2019; Sabra et al. 2016). On the other hand, the addition of butyrate at the initial stage of cultivation, either as a sole supplement (Figure 6), or in combination with acetate (Figure S2), induced an earlier synthesis of CA production in *M. cerevisiae* broth. Interestingly, the metabolic flux analyses depicted in Figure 5 and in Figure S1, revealed that butyric acid production was mainly synthesised by the RBO pathway, while major fluxes of CA production are through the FAS pathway. The observed elevated fluxes of CA synthesis via the FAS pathway may provide a theoretical basis for the metabolic divergence between strains that produce MCFAs and those that predominantly accumulate butyric acid without further elongation, as observed in *Clostridium pasteurianum* (Sabra et al. 2014). These metabolic differences can be elucidated through further use of ^13^C MFA, which will be the focus of future work in our laboratories (Bommareddy et al. 2014; Nicolae et al. 2014).

**FIGURE 8:**
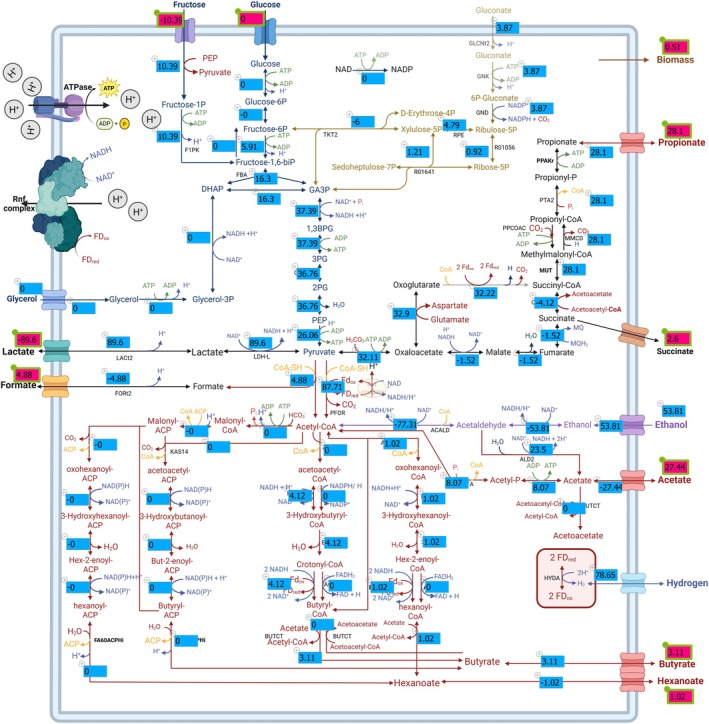
Metabolic flux analysis of *M. cerevisiae* metabolism on lactate and butyrate showing an inconsistent scenario predicting ethanol production to resolve redox imbalance. Model constraints were based on measured metabolite uptake and secretion rates in mmol/g biomass *h (mean values from Figure 7).

Lactate is a significant end‐product of glycolysis and serves as an energy substrate for numerous anaerobic bacteria, including *Acetobacterium woodii* , *Veillonella critici* and *Desulfotomaculum reducens* (Weghoff et al. 2015; Dietz et al. 2013; Dai et al. 2024, 17; Sabra et al. 2013; Prabhu et al. 2012). lactate based chain elongation was reported in *M. hexanoica* (Kang et al. 2022) and M. elsendii (Prabhu et al. 2012; Kim et al. 2020). While lactate production is associated with enhanced initial growth of *M. cerevisiae* (Figures 1 and 2), its specific contribution to chain elongation remains unclear. In fact, as demonstrated in Figure 7, co‐fermentation involving lactate, butyrate, and fructose did not result in CA production, even in the presence of butyrate. Furthermore, lactate assimilation resulted in the production of propionate and acetate, exhibiting a metabolic profile analogous to that observed in cultures of *Veillonella criticae* (Sabra et al. 2013). Similarly, MFA was conducted during the initial cultivation phase until lactate was fully consumed, and the measured metabolite concentrations, expressed in mmol/g_DW_/h, were used to constrain the simulation of the metabolic network depicted in Figure 1. Neverthesless, the model failed to align with the experimental data shown in Figure 7, yielding feasible solutions only under conditions that permitted ethanol production (Figure 8). This discrepancy caused by a marked imbalance in reducing equivalents, and the NADH produced in reactions producing pyruvate (LDH), 1,3‐biphosphoglycerate (GAPD), acetate (ALD2) and oxaloacetate (MDH) can be balanced in ethanol‐producing reactions through acetaldehyde dehydrogenase (ACALD) and alcohol dehydrogenase (ADH1). Together, these two reactions represent over 86% of the total NADH utilisation (see Figure S3).

**FIGURE 9:**
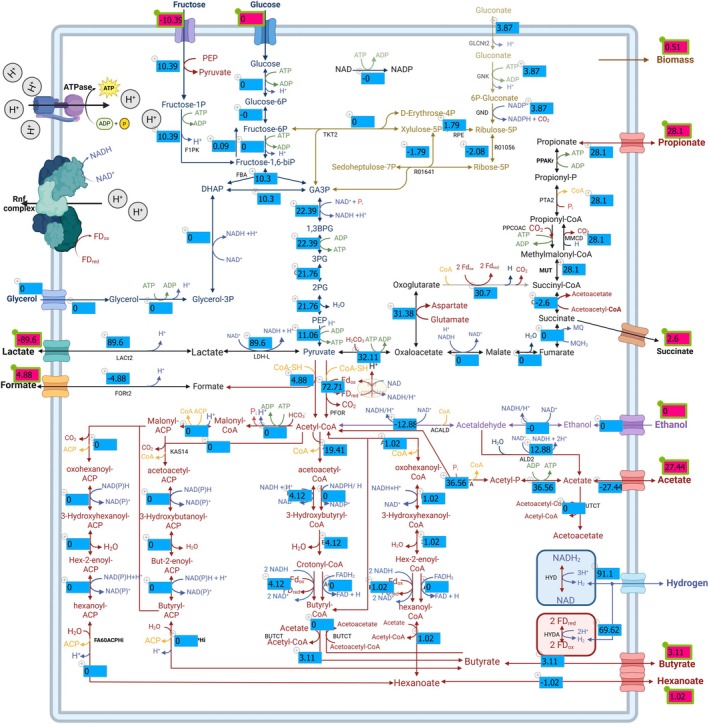
MFA of M. cerevisiae cultivated in PF medium supplemented with lactate and butyrate. The metabolic network was modified to include a NADH‐dependent hydrogenase reaction. Experimental flux values derived from Figure 7 were applied as model constraints.

Although *M. cerevisiae* possesses numerous transcripts associated with lactate metabolism, including L‐lactate dehydrogenase and L‐lactate permease (Bergsveinson et al. 2017), its growth on lactate remains energetically challenging because of the elevated redox potential of the pyruvate/lactate pair, which necessitates an energetically uphill electron transfer from lactate to NAD+ (Weghoff et al. 2015). Such thermodynamic constraints contribute to the observed *in silico* imbalance in reducing equivalents during lactate utilisation (Figure 8). Indeed, various bacterial species have evolved mechanisms that enable efficient reoxidation of reducing equivalents during lactate metabolism (Weghoff et al. 2015; Soto‐Cruz et al. 2002; Louis et al. 2022). Among these, hydrogen production is of particular note as a highly effective electron sink under anaerobic conditions. Notably, *M. cerevisiae* possesses the hydA gene (WP_074501648.1) responsible for oxidising reduced ferredoxin and subsequently producing hydrogen. Although HydA is typically considered to be part of the HydABC complex, BLAST analyses revealed that the other two subunits are absent from the *M. cerevisiae* genome. However, analysis of the *M. cerevisiae* strain revealed the presence of a NADH‐dependent [FeFe] hydrogenase, classified within group A6 (KMO86138.1). This gene was not annotated by the RAST toolkit, reflecting the more general issue of incomplete pathway annotation in microbial genomes highlighted by (Griesemer et al. 2018). As shown in Figure 9, incorporating the NADH‐dependent hydrogenase reaction into the metabolic model made it feasible, providing strong evidence that NADH reoxidation in *M. cerevisiae* is primarily driven by hydrogen production rather than ethanol formation.

In this study, we present the first genome‐scale metabolic reconstruction of *M. cerevisiae* DSMZ 20462 central metabolism, encompassing 109 reactions and 93 metabolites. The model was manually curated and validated against experimental data, providing a comprehensive depiction of the organism's core metabolic functions and establishing a foundation for future in silico exploration of central metabolic pathways and medium‐chain fatty acid (MCFA) biosynthesis in this beer spoilage strain. The experimental validation process highlighted the significance of media supplements in optimising caproate production. It was also shown that lactate, which has been identified as a key carbon source for caproate synthesis in different Megasphaera strains (Kang et al. 2022; Wang et al. 2025), did not contribute to caproate formation in M. cerevisiae. Overall, this study provides fundamental insights that enhance our understanding of beer spoilage by *M. cerevisiae* .

### 4.1 Limitations of the Study

In silico reconstruction of the central metabolism of *M. cerevisiae* DSMZ 20462 suggests that the FAS pathway is crucial for CA formation, while butyric acid production is primarily driven by the RBO pathway, with minor inputs from the FAS pathway. The differential activation of elongation routes may explain the metabolic differences observed between MCFA‐producing bacteria and those that terminate at butyrate. However, this interpretation remains preliminary and requires further investigation. A central aim of our future work is therefore to perform a comparative multi‐omics analysis across diverse MCFA‐producing strains and well‐characterised butyrate‐forming bacteria that do not engage in further chain elongation. Importantly, future studies will incorporate direct quantitative measurements of gas analysis such as H_2_ and CO_2_, rather than relying solely on qualitative, redox‐based inference of hydrogen production. This integrated approach will enable a more robust evaluation of pathway activity in *M. cerevisiae* and could therefore provide valuable foundational insights for understanding and potentially controlling beer spoilage in industrial brewing environments.

## Author Contributions

**Wael Sabra:** conceptualization, investigation, writing – original draft, methodology, validation, visualization, writing – review and editing, data curation, supervision. **Sonia Villotti:** investigation, methodology, validation, data curation. **An‐Ping Zeng:** writing – review and editing, funding acquisition, resources, project administration. **Ahmed M. Haddad:** conceptualization, investigation, writing – review and editing, validation, methodology. **Joachim Fensterle:** writing – review and editing, resources, visualization.

## Conflicts of Interest

The authors declare no conflicts of interest.

## Supporting information


**File S1:** Supporting Information.

**Figure S1:** Metabolic flux analysis of *Megasphaera cerevisiae* cultivated on fructose and butyrate. (A) During the initial phase, constraints were applied to both fructose and butyrate uptake in the metabolic model. (B) In the subsequent phase, only fructose uptake was used as a model constraint.
**Figure S2:** Growth and acid production of *Megasphaera cerevisiae* in a pH‑controlled bioreactor using semisynthetic medium supplemented with acetate (1.25 g/L) and butyrate (1.75 g/L).
**Figure S3:** NADH‐producing and ‐consuming reactions derived from the metabolic model depicted in Figure S1, using experimental data with lactate supplementation. LDH: lactate dehydrogenase; GAPD: Glyceraldehyde‐3‐phosphate dehydrogenase; ALD2: Acetaldehyde:NAD+ oxidoreductase; MDH: malate dehydrogenase; ACALD: Acetaldehyde dehydrogenase; ADH1: Alcohol dehydrogenase; ACOAD1 Butanoyl‐CoA:2‐oxidoreductase; ACAD2: Acyl‐CoA dehydrogenase (hexanoyl‐CoA); HACD1: (S)‐3‐Hydroxybutanoyl‐CoA:NAD+ oxidoreductase; HACD2: 3‐hydroxyacyl‐CoA dehydrogenase (3‐oxohexanoyl‐CoA).

## Data Availability

The data that supports the findings of this study are available in the supplementary material of this article, or from the corresponding author upon request.
