# Supplementary material for: Metabolic Flux and Growth Profiling of Megasphaera cerevisiae for Medium‐Chain Fatty Acid Synthesis
Source: Environ Microbiol Rep. 2026 Jul 24;18(4):e70390. doi: 10.1111/1758-2229.70390 (PMC13400456; doi:10.1111/1758-2229.70390)
Supplement: Supplementary file 2 — Figure S1: Metabolic flux analysis of Megasphaera cerevisiae cultivated on fructose and butyrate. (A) During the initial phase, constraints were applied to both fructose and butyrate uptake in the metabolic model. (B) In the subsequent phase, only fructose uptake was used as a model constraint. Figure S2: Growth and acid production of Megasphaera cerevisiae in a pH‑controlled bioreactor using semisynthetic medium supplemented with acetate (1.25 g/L) and butyrate (1.75 g/L). Figure S3: NADH‐producing and ‐consuming reactions derived from the metabolic model depicted in Figure S1, using experimental data with lactate supplementation. LDH: lactate dehydrogenase; GAPD: Glyceraldehyde‐3‐phosphate dehydrogenase; ALD2: Acetaldehyde:NAD+ oxidoreductase; MDH: malate dehydrogenase; ACALD: Acetaldehyde dehydrogenase; ADH1: Alcohol dehydrogenase; ACOAD1 Butanoyl‐CoA:2‐oxidoreductase; ACAD2: Acyl‐CoA dehydrogenase (hexanoyl‐CoA); HACD1: (S)‐3‐Hydroxybutanoyl‐CoA:NAD+ oxidoreductase; HACD2: 3‐hydroxyacyl‐CoA dehydrogenase (3‐oxohexanoyl‐CoA). [file EMI4-18-e70390-s001.docx]

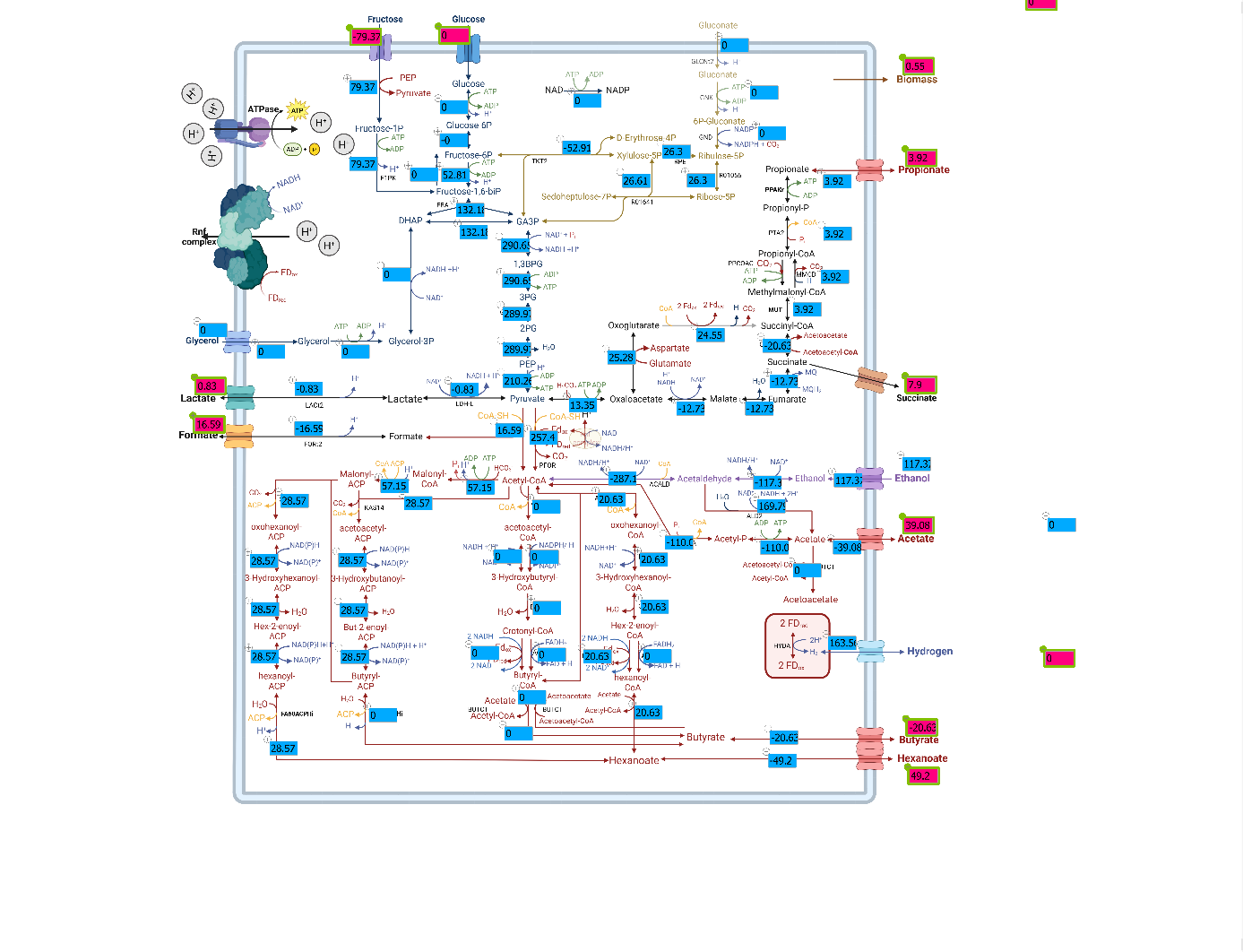


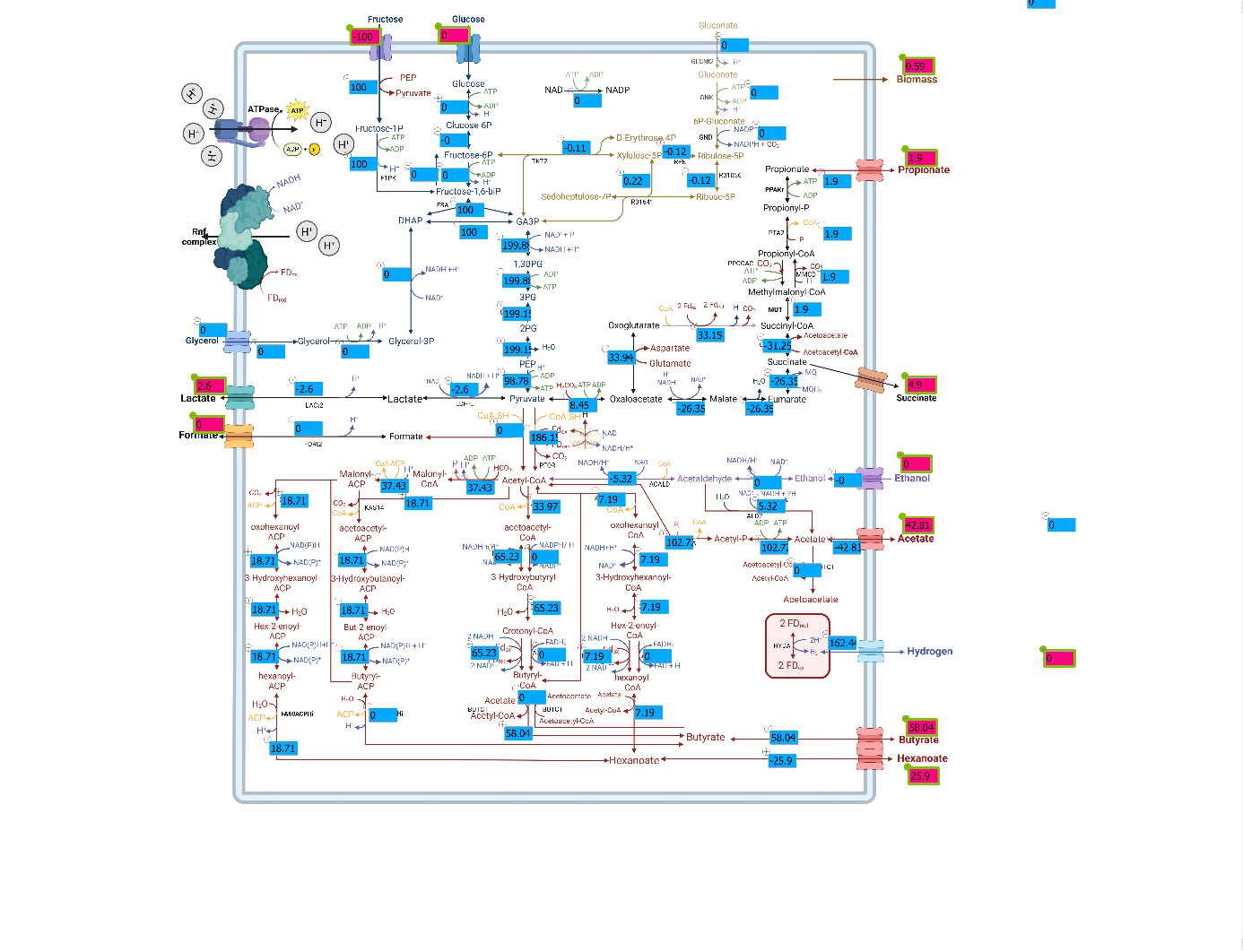


**Figure 1 (Supplementary).** Metabolic flux analysis of Megasphaera cerevisiae cultivated on fructose and butyrate. (A) During the initial phase, constraints were applied to both fructose and butyrate uptake in the metabolic model. (B) In the subsequent phase, only fructose uptake was used as a model constraint.

**Figure 2 (Supplementary)**: Growth and acid production of Megasphaera cerevisiae in a pH‑controlled bioreactor using semisynthetic medium supplemented with acetate (1.25 g/L) and butyrate (1.75 g/L).


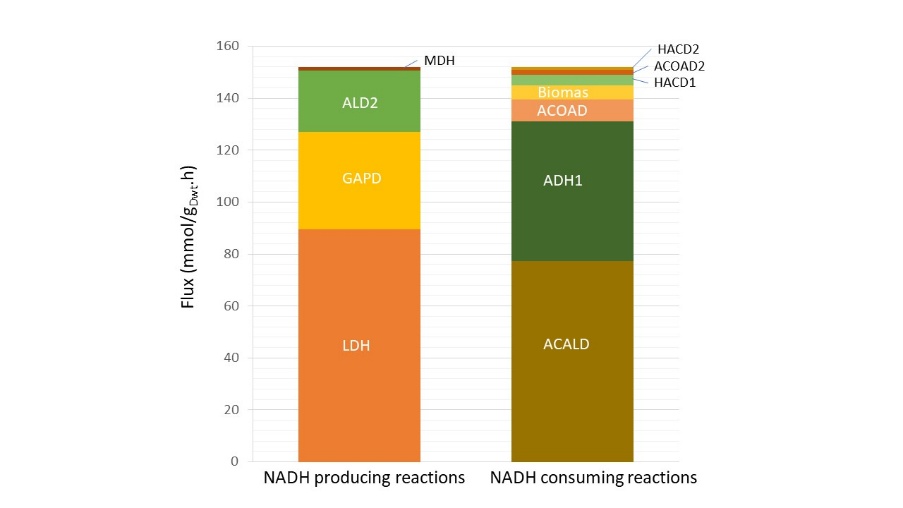


**Figure 3 (Supplementary).** NADH-producing and -consuming reactions derived from the metabolic model depicted in Figure 1, using experimental data with lactate supplementation. LDH: lactate dehydrogenase; GAPD: Glyceraldehyde-3-phosphate dehydrogenase; ALD2: Acetaldehyde:NAD+ oxidoreductase; MDH: malate dehydrogenase; ACALD: Acetaldehyde dehydrogenase; ADH1: Alcohol dehydrogenase; ACOAD1 Butanoyl-CoA:2-oxidoreductase; ACAD2: Acyl-CoA dehydrogenase (hexanoyl-CoA); HACD1: (S)-3-Hydroxybutanoyl-CoA:NAD+ oxidoreductase; HACD2: 3-hydroxyacyl-CoA dehydrogenase (3-oxohexanoyl-CoA)
